# Predicting Postoperative Vomiting for Orthopedic Patients Receiving Patient-Controlled Epidural Analgesia with the Application of an Artificial Neural Network

**DOI:** 10.1155/2014/786418

**Published:** 2014-08-05

**Authors:** Cihun-Siyong Alex Gong, Lu Yu, Chien-Kun Ting, Mei-Yung Tsou, Kuang-Yi Chang, Chih-Long Shen, Shih-Pin Lin

**Affiliations:** ^1^Department of Electrical Engineering, School of Electrical and Computer Engineering, College of Engineering, Chang Gung University, Taoyuan 333, Taiwan; ^2^Portable Energy System Group, Green Technology Research Center, College of Engineering, Chang Gung University, Taoyuan 333, Taiwan; ^3^Department of Biomedical Engineering, College of Basic Medical Sciences, China Medical University, Shenyang, Liaoning 110001, China; ^4^Department of Anesthesiology, Taipei Veterans General Hospital and National Yang-Ming University, No. 201, Section 2, Shi-Pai Road, Taipei 112, Taiwan; ^5^Section of Anesthesiology, Ton-Yen General Hospital, Hsinchu 302, Taiwan

## Abstract

Patient-controlled epidural analgesia (PCEA) was used in many patients receiving orthopedic surgery to reduce postoperative pain but is accompanied with certain incidence of vomiting. Predictions of the vomiting event, however, were addressed by only a few authors using logistic regression (LR) models. Artificial neural networks (ANN) are pattern-recognition tools that can be used to detect complex patterns within data sets. The purpose of this study was to develop the ANN based predictive model to identify patients with high risk of vomiting during PCEA used. From January to March 2007, the PCEA records of 195 patients receiving PCEA after orthopedic surgery were used to develop the two predicting models. The ANN model had a largest area under curve (AUC) in receiver operating characteristic (ROC) curve. The areas under ROC curves of ANN and LR models were 0.900 and 0.761, respectively. The computer-based predictive model should be useful in increasing vigilance in those patients most at risk for vomiting while PCEA is used, allowing for patient-specific therapeutic intervention, or even in suggesting the use of alternative methods of analgesia.

## 1. Introduction

Vomiting is one of the most frequent adverse effects of patient-controlled epidural analgesia (PCEA) with reported incidence of 3.1 to 34% [1–4]. The clinical importance of this side effect has been shown in several studies which proved that vomiting episodes clearly correlated patients' satisfaction with PCEA and it can reduce the percentage of PCEA for use. Routine medications to prevent vomiting are not recommended for several reasons including potential side-effects of antiemetic drugs, lack of increased patient satisfaction, and economic reasons [5, 6]. Thus, identification of patients with high risk of vomiting related to PCEA would be an important step in the rational selection of patient-controlled epidural analgesia and adoption of therapeutic interventions.

Artificial neural networks are pattern-recognition tools that can be used to detect complex patterns within data sets. In recent years ANN has been widely applied in computer-aided diagnosis [7, 8], outcome prediction [9, 10], and signal processing [11, 12]. A good predictive model for postoperative nausea and vomiting (PONV) helps us do risk classification and management. No studies have ever been reported to investigate this topic using ANN model, especially in the PCEA case. If the predicting model among the PCEA agents and risk factors can be established, we could adjust baseline infusion rate of PCEA on the basis of individual conditions. On the other hand, to explain the high possibility of vomiting to high-risk patient according to the predicting model before the PCEA is used can also reduce the potential discomfort and then their anxiety, thereby increasing the satisfaction with our service as well. Therefore, we conducted this retrospective study to develop the ANN-based model to predict patients with high risk of vomiting during PCEA. Furthermore, we compared the predictive performance of the ANN model to the prediction of logistic regression model.

## 2. Materials and Methods

### 2.1. Study Population

This retrospective study was conducted at Taipei Veterans General Hospital with the approval of our Institutional Review Board (VGHIRB No.:96-10-07A). We collected data on surgical patients consenting to epidural analgesia from Jan. to March 2007. All patients underwent operations involving lower extremities with postoperative PCEA included. Patients with missing demographic data were eliminated from the list. Epidural catheters were placed before the operations corresponding to the dermatomal level of surgical incision. 18-gauge Tuohy needle and 20-gauge epidural catheter were employed. A loss-of-resistance technique was used to identify the epidural space, and the epidural catheter was placed at a location 5–7 cm into the epidural space. The catheter was affixed with skin adhesive along the patient's back. All epidural catheters were tested for adequate function and intrathecal or intravascular migration was ruled out before surgery. Patients received a standardized combined spinal-epidural anesthesia with intrathecal hyperbaric 0.5% bupivacaine 12~15 mg and a 6~10 mL epidural loading dose of bupivacaine (0.25%) with fentanyl (5 *μ*g/mL) during operation.

On arrival in the postanesthesia care unit, a patient-controlled analgesia device (Aim plus system, Abbott Laboratories, North Chicago, IL, USA) was connected to patient's epidural catheter. An analgesic solution of bupivacaine (0.0625%) and fentanyl (1 *μ*g/mL) was used for all patients from published recommended doses. 10–12 initial PCEA settings were a baseline infusion of 3~5 mL/h with a PCEA bolus of 2 mL and lockout interval of 20~30 min. Inadequate analgesia (verbal pain score ≧ 5, where 0 = no pain and 10 = most intense pain imaginable) was treated with a 5 mL loading dose of the infusion mixture followed by an increase in the baseline infusion of 1~2 mL/h. After setup of the patient-controlled analgesia (PCA) device, the continuous infusion and the cumulative dose recording were started. All patients were visited once a day by the PCA team staff in the morning or afternoon and whenever clinically necessary. Any complaint about numbness, nausea, vomiting, pruritus, or other adverse effects related to PCEA was treated with decreasing 1~2 mL continuous dose based on the severity and the events were recorded on the PCEA charts.

The following patient's variables were included in the study for model construction and performance evaluation:

1. patient-related variables including age, gender, height, weight, and body mass index (BMI);
2. surgery and PCEA variables including type of surgery, bolus epidural dose of PCA, epidural catheter insertion level, and epidural catheter length in the epidural space;
3. depending variable of vomiting, a binary variable defined as patients who experienced the forceful expulsion of gastric contents through the mouth or nose during postoperative three days.

### 2.2. Logistic Regression (LR)

Data analysis and statistics were performed using SAS software (V9.2; SAS Institute Inc., Cary, NC, USA.). We calculated mean value, standard deviation, and 95% confidence interval as metric variables. Categorical variables were assessed for a significant association by Chi-square statistics. Forward selection algorithm was used for the variable selection. At each step, independent variables not yet included in the equation were tested for possible inclusion. The variable with the strongest significant contribution to improve the model was included in the equation. Variables already included in the logistic regression equation were tested for exclusion on the basis of the probability of a log likelihood test ratio. The analysis ended when no further variables were available for inclusion or exclusion. Logistic regression analyses were used for odds ratio (OR) estimation. After univariate analysis, we selected nine variables according to the related literatures and clinical experiences to the coefficients (*β*) of these variables. On the basis of the results, the probability of vomiting may be estimated with the logistic equation.

### 2.3. Artificial Neural Network (ANN)

We used the NeuroSolutions for Excel (Version 5.0, NeuroDimension Inc.) to develop the ANN model. A multilayer perception (MLP) ANN was used to train the predictive model. After the training process was finished, the final ANN model was tested with the remaining patients (*n* = 49), who were not selected for training and whose outcome regarding occlusion was unknown to the ANN, from the original sample. The ANN construction consisted of one input layer, one hidden layer, and one output layer. We tested eight, ten, and twelve neurons in the hidden layer and one neuron in output layer. There were 9 parameters chosen as input variables according to the related literatures and clinical experiences. The mean square errors (MSE) of each iteration were computed and averaged, and we selected the final ANN model by the criteria of which MSE was closest to the average MSE (average of MSE of all ANN models) (see Table 1).

### 2.4. Performance Evaluation

Model performance was evaluated with holdout method. Data were randomly selected where 75% were for model construction and the remaining 25% were used as test set for validating the predictive performance. The test data set was used to evaluate predictive performance. The discriminating power of these prediction models can be measured by the receiver operating characteristic (ROC) curves. To provide an unbiased estimation of model's discrimination, these values have to be calculated from a test set not used in the model building process. ROC analysis estimates a curve that describes the inherent trade-off between sensitivity and specificity of a prediction tool. Discriminatory power is measured by the area under ROC curve (AUC). AUC represents a common measure of sensitivity and specificity over all possible thresholds.

## 3. Results

All patients were classified into vomiting and nonvomiting groups according to their response to PCEA. Continuous data are presented as mean with standard deviation (SD) and categorical data are expressed as count with percentage. Independent* t*-test or Chi-square test was used to compare patients' characteristics and variables related to PCEA usage of the two groups. The demographic data and characteristics related to PCEA usage were shown in Table 2. The overall incidence rate of vomiting for orthopedic patients receiving PCEA was about 30.6% (49.0% for female and 7.7% for male).

The results of logistic regression analysis are summarized in Table 3. There are three variables included in the final logistic regression model: gender, catheter length in epidural space, and TKA. The probability of vomiting can be calculated by the following logistic equation:

Probability = 1/1 + *e* ^−*β*^, with *β* = 2.658 + 2.363*(Gender) − 0.712*(Catheter  length  in  epidural  space) − 0.941*(TKA). Table 3 shows the unadjusted and adjusted OR of some potential risk factors related to vomiting induced by PCEA. By univariate analyses, female sex and catheter length in epidural space are associated with vomiting during the course of PCEA. Gender is the most significant factor related to vomiting (unadjusted OR = 8.143, 95% CI: 2.710–24.463). In contrast, catheter length plays a protective role in PCEA-related vomiting in univariate analysis. Other factors did not have statistically significant influence on vomiting. After forward model selection, female sex is still the most significant risk factor related to the PCEA-induced vomiting. The adjusted OR and its 95% CI of female gender are 10.621 and 3.135–35.975, respectively. Other risk factors after adjustment included catheter length in epidural space (OR = 0.490, 95% CI: 0.308–0.782) and TKA surgery (OR = 0.390, 95% CI: 0.152–1.000).

**Figure 1:**
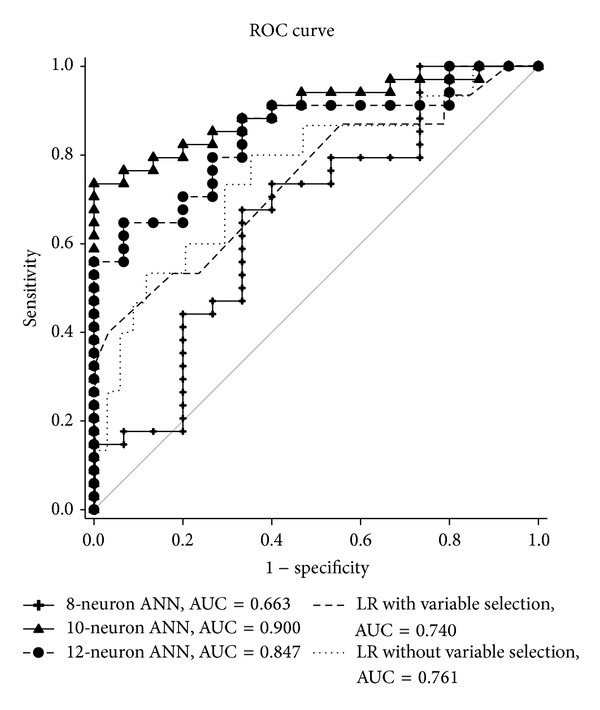
ROC curve of logistic regression and ANN.

The ANN has better AUC value in predicting vomiting (Table 4). We found ANN using 10 nodes has best AUC value than 8 nodes and 12 nodes. The area under ROC curves of ANN and LR models was 0.900 and 0.761, respectively. The receiver operative characteristic (ROC) curves were plotted in Figure 1. It revealed that the ANN model has better discriminating power than the LR model to identify the patient with high risk to develop vomiting while receiving PCEA after orthopedic surgery.

## 4. Discussion

Since high quality PCEA has been playing an important role in postoperative orthopedic patient care, a good predicting tool to avoid complications, especially vomiting, is very important. The incidence of vomiting may differ due to many factors. For example, morphine-based PCEA may induce more vomiting than fentanyl-based one [13]. Good predicting model associated vomiting in PCEA patient is necessary to identify susceptible subjects and then prevention strategy could be proposed in advance to reduce the incidence of vomiting. In our study the ANN and LR models demonstrated the power in detecting whether vomiting occurred after using PCEA. The ROC curves were plotted to summarize the findings of the multivariate analysis.

The ANN had better value of AUC than LR in this study. Computer-based medical decision support systems have recently been studied and used clinically for medical diagnosis and improvement of patient care [14]. The ANN used in this study can be easily used with any standard desktop computer. Nevertheless, the ANN can be easily developed in any institution for local use. Therefore, the ANN appears to be a very suitable model for clinicians to use in putting rational and cost-effective antiemetic treatments into practice. In this study, we use the 9 variables relative to PCEA induced vomiting to construct the ANN model. The ANN model with the 9 variables presented a good predictive performance superior to the LR model which was developed from the same 9 parameters. In clinical practice, the ANN model which needs only 9 parameters would be easier to use and probably acceptable. On the other hand, the knowledge of these risk factors and the computer-based predictive models should be useful in increasing vigilance in those patients most at risk for vomiting, in allowing for patient-specific therapeutic intervention [9, 15, 16], or even in suggesting the use of preventive strategy for them. For example, if a patient is predicted to have high risk of vomiting, we should use prophylactic antiemetics, for example, metoclopramide, droperidol, can be prescribed before initiation of PCEA regimen. Other antiemetics, such as ondansetron and dexamethasone, can also be used as combination of management according to the risk of vomiting. ANN model can be served as a risk classification tool to manage the PCEA-related vomiting more efficiently.

In our LR models, all of the predictors were chosen to find the possible risk factors by fitting a logistic regression a stepwise forward selection procedure (*P* < 0.05 to enter). Several potential factors associated with vomiting were identified. Through the forward logistic model selection, we researched into the factors including the female gender, catheter length in epidural space, and TKA surgery. The finding that the female is risk factor of vomiting is compatible with Tsui et al. [1]. Since female sex is a risk factor of vomiting induced by PCEA, preventive strategy may be considered for them. But why the longer epidural catheter length is the protective factor? We had known that longer epidural catheter length insertion increased the risk of intravenous insertion, intrathecal migration, knotting, or unilateral sensory analgesia [17]. Although this finding is remarkable, the exact mechanism is not clear. In other words, the relation between the incidence of vomiting after PCEA was used and the epidural catheter length needs further study to evaluate. Among the several types of orthopedic surgery enrolled in this study, we found that patients who received the total knee replacement are risky for vomiting while they received PCEA. The possible reasons are still unknown. However, this effect is minor in the final regression model and we cannot have a definite conclusion of this effect in the current study. To the best of our knowledge, there is still no study about the relation between the epidural catheter length and the incidence of vomiting.

There are some limitations in our studies. First, the case number we enrolled is relatively little for a data mining study; further cases should be collected to increase the power of analyses. Second, more valuable variables, for example, no smoking, anxiety, and history of PONV, should be included in the analysis. Increasing the case numbers and variables may increase the predicting model AUC. Third, because the purpose of our predictive model was to provide a simple method that can be used easily in clinical setting, we grouped all orthopedic operations with PCEA used. We did not classify the data into a more detailed way. This may lose some procedure-specific information.

In conclusion, the ANN and LR models which were developed by the parameters available before PCEA was used demonstrated the power in detecting whether vomiting occurred after PCEA was used. Our study has some clinical implications. Firstly, when the models were applied in clinical practice, we can identify the patient with high risk of vomiting before PCEA is performed. Secondly, we could undertake some therapeutic interventions to prevent the occurrence of vomiting or consider if other analgesic techniques could be used. Preventive strategy can be provided in advance to reduce the incidence of vomiting. Thirdly, we can use this individualized model to explain the risk of vomiting to patient receiving PCEA.

## Figures and Tables

**Table 1:** Variables used for training of the LR and ANN model.

Variable	Coding
Age	Years
Gender	1: female; 0: male
Height	cm
Weight	kg
BMI	Body mass index
Length (catheter length in the epidural space)	cm
Bolus dose	mL
Total knee replacement (TKA)	0: not TKA∗; 1: TKA
Epidural level (insertion site of EA catheter)	0: above L4; 1: below L4

*Other lower extremities surgery.

**Table 2:** Patient characteristics.

	Value (*n* = 195)
Age	69.6 ± 12.3
Catheter length in epidural space (cm)	6.9 ± 0.9
Height (cm)	158.2 ± 9.3
Weight (cm)	65.3 ± 11.7
BMI	26.1 ± 4.1
Bolus dose	2.0 ± 0.3
Gender	
Female	104 (53.3%)
Male	91 (46.7%)
Operation type	
Total knee replacement	133 (68.2%)
Others	62 (31.8%)
Epidural level	
L2-3	37 (19.0%)
L3-4	142 (72.8%)
L4-5	16 (8.2%)

Parametric data were shown as mean with SD.

Categorical data were shown as count and percentage.

**Table 3:** The unadjusted and adjusted OR of some potential risk factors related to vomiting induced by PCEA.

	Unadjusted OR	95% CI		*P* value	Adjusted OR	95% CI		*P* value
		Lower	Upper			Lower	Upper	
Gender (female)	8.143	2.710	24.463	<0.001	10.621	3.135	35.975	<0.001
Age (year)	1.014	0.986	1.043	0.338				
Total knee replacement (TKA)	0.889	0.414	1.910	0.1763	0.39	0.152	1.000	0.049
Epidural level	0.361	0.118	1.103	0.074				
Length (cm)	0.498	0.325	0.763	0.001	0.490	0.308	0.782	0.003
Height (cm)	0.960	0.919	1.003	0.069				
Weight (kg)	0.973	0.943	1.004	0.086				
Bolus dose (mL)	0.506	0.120	2.123	0.352				
BMI (kg/m^2^)	0.969	0.893	1.051	0.443				

**Table 4:** Comparison of predictive performance of logistic regression (LR), and artificial neuronal network (ANN) using test dataset.

	ANN (8 neurons)	ANN (10 neurons)	ANN (12 neurons)	Logistic regression (without variable selection)	Logistic regression (with variable selection)
AUC	0.663	0.900	0.847	0.761	0.740
Standard error	0.089	0.043	0.055	0.079	0.083

AUC, area under ROC curve.
